# Development and Test of Geogrid with Distributed Deformation Monitoring Function

**DOI:** 10.3390/ma17020331

**Published:** 2024-01-09

**Authors:** Jiong Zhang, Yi Li, Bowen Meng, Jie Ding, Rui She, Shipu Ren, Qifang Liu

**Affiliations:** 1 School of Civil Engineering, Shandong University, No. 17923 Jingshi Road, Jinan 250000, China; 202135029@mail.sdu.edu.cn (Y.L.);; 2 Shandong Huajian Engineering Testing Company Limited, Luguang Technology Industrial Park, Keyuan Road, High-Tech Zone, Jinan 250000, China

**Keywords:** 3D printed geogrid, distributed deformation monitoring, electrical resistance

## Abstract

In recent years, there is a growing demand for materials that can both improve the mechanical properties of structures and carry out health monitoring and risk warning. In this case, in order to realize distributed deformation monitoring, a new method of making geogrid by 3D printing technology is proposed. The grille rib is made by embedding the conductive polymer (ground carbon fiber as conductive filler) into the insulating shell (PLA material) in the specified path, and then the rib is vertically crossed into each other to form a grille sample. In order to study the distributed deformation monitoring function of this grid, a manual push–pull testing machine was used to conduct a load–unload experiment to analyze the change rule of resistance on the grid plane. The following conclusions were obtained: the closer the ribs are to the load bearing point, the greater the change in resistance, and conversely, the farther the ribs are from the load bearing point, the smaller the change in resistance. Depending on the geogrid network characteristics, the electrical resistance distribution on the geogrid plane can be obtained by superimposing the resistance values of the horizontal and longitudinal ribs, then the location and the magnitude of deformation can be estimated. Additionally, this study carried out numerical simulation of the grid model based on ANSYS 15.0 software and compared with the loading experiment results to verify that the force deformation position can be retrieved through the change of resistance.

## 1. Introduction

There is a growing demand for materials that can both improve the mechanical properties of structures and carry out health monitoring and risk warning. Geogrids have been extensively utilized to fulfill reinforcement of geotechnical structures such as slopes, dikes, dams, railways, embankments, and landfills [1,2,3,4] By integrating sensing materials, which are sensitive to parameters such as strain or temperature, into the geogrid in a variety of methods, it can send warning signals before the structures fail.

Strain gauges are earlier examples used to measure strains in geosynthetics where sensing is achieved by attaching these devices to a geosynthetic layer in critical positions [5,6,7,8] Strain gauges must be calibrated during the use period, but calibration factors are typically not accurate due to the local stiffening effect of the bonding assembly and interference with the soil’s surroundings [9] The strain gauges adhered on geogrids were susceptible to falling off, and the gauges might be affected by moisture or electromagnetic interference when being used in harsh environments, which provided unreliable strain measurement results [10].

Fiber sensors have been widely used for measuring some physical parameters such as pressure, strain, etc. [11,12,13,14,15], because of numerous advantages, such as electromagnetic interference immunity, lightweight, smaller size, remote operation, and excellent sensitivity. Additionally, a fiber optic sensors-based sensing system can be used for distributed/quasidistributed real-time monitoring, and then delivering all sensing information through one single fiber optic cable. Sensor-enabled geogrid (SEGG) technology has been introduced in the past few years as a new category of geogrid products that possess embedded strain-sensing capability in addition to their conventional reinforcement/stabilization function in geotechnical and transportation applications.

Wang et al. [16] developed smart geogrids embedded with fiber Bragg-grating (which is a newly developed sensing element for detecting strain, temperature, and other physical quantities in recent years), and analyzed strain distribution and the strain transfer characteristics of geogrids by using finite element simulation. They conducted a series of experiments to verify that the smart geogrids exhibit good consistency in strain measurement and can accurately measure the local strain of geotechnical structures with small dimensions. Additionally, a deformation reconstruction technique has been investigated [17] which enables the smart geogrid to evaluate the deformation fields of the key areas in geotechnical structures. However, silica-based fiber sensors are limited in application due to their low break-down strain of only 1%, and they are fragile, requiring stricter packaging methods and installation procedures.

To overcome the shortcomings mentioned above, the researchers made in-depth studies on the development and application of polymer fiber optic (PFO) sensors with excellent fracture toughness and flexibility [18,19,20,21]. Although PFO has been proven to have robust sensor capabilities, it is vulnerable to unnecessary loss of strength, such as bending loss or connector loss, as well as power supply and change.

Some studies have shown that electrically conductive polymer composites (ECPCs) can be obtained when good conductors of electrical current (carbon black, graphite powder, carbon fiber, microparticles of metals) are implanted into an insulating polymer matrix [22,23,24,25]. As the concentration of conductive fillers reaches sufficient value, a continuous conductive network can be formed, which provides the electrons a relatively low-resistance electrical path to move freely [26,27]. Deformation of conductive composites under external forces results in a change in their electrical resistivity, resulting in a change in volume electrical resistivity. However, carbon-based polymer composites have the characteristics of light weight, good mechanical properties, good conductivity, and low cost. These properties provide new ideas for the development of geosynthetics. Das et al. [28] studied the variation of electrical resistivity of carbon black and short carbon rubber composites at constant strain rate. It was found that electrical resistivity was irreversibly altered in extension–retraction cycles. Mohd Radzuan investigated the potential use of milled carbon fiber as a conductive filler in a composite and chose carbon nanotube (CNT) and carbon black (CB) as secondary fillers, which significantly improved the conductivity of composites.

Hatami et al. [29] pioneered a new geosynthetic by adding CNT and CB to high-density polyethylene and polypropylene (PP), which can measure the tensile strain based on the piezoresistivity. This new geosynthetic enables the measurement of enhanced strain at larger data points without considering the adverse structural effects of instruments buried in the soil. Hatami et al. [30] also developed a new generation of geosynthetics made of coated polyester yarn, and the coating is made of carbon black-filled PVC. However, the SEG layer is directly in contact with the soil, the electric circuit is affected by the moisture content and electrolytes, and the research on solving these problems is very limited.

Cui et al. [31] developed a new smart geosynthetic named sensor-enabled geo-belt (SEGB). The SEGB of high-density polyethylene (HDPE) filled with carbon black (CB) was fabricated by both the industry and the laboratory. Hot pyrocondensation pipes (HPPs) were used to protect the SEGB against the influence of water. The effects of thermal oxidation, ultraviolet radiation, acid–base corrosion, and cyclic loading on SEGB properties were then tested [32,33] However, the tensile strength of SEGB and the friction between SEGB and the soil after sealing were reduced, and the manufacturing difficulty was increased. The strain–conductivity response may be complex because the whole geo-belt is conductive and contacts each other. In order to realize distributed monitoring, it is necessary to arrange the conductive adhesive on the SEBG surface according to the selected distance and use pairs of wires to connect. However, this will lead to too much test data, and it is difficult to measure; the thickness of the wire should be well controlled, otherwise it will have an adverse effect on the interface between the geo-belt and soil.

Zhang et al. [34] embedded the conductive polymer of PLA into the insulating material to make smart geo-belt through 3D printing technology, and carried out a series of tests to verify its good self-sensing performance. Based on the above research, 3D-printed connectors were used in this study to connect the geo-belt horizontally and longitudinally to form a geogrid with distributed deformation monitoring function, which is of practical significance for the safety of rock and soil structures. In addition, wires were welded at two ends to measure the resistance. The influence of moisture content on the circuit can be avoided, and the ribs are completely insulated from each other. In addition, loading–unload experiments were carried out in this study, and the characteristics of resistance change on the geogrid plane was analyzed. The location of deformation area can be determined through the resistance distribution on the grid plane, and distributed measurement is achieved with no need to lay out a mass of wires, which simplifies the monitoring method and avoids the complex problems of construction. What is unique is that the results of the numerical simulation method in this study further support the feasibility and accuracy of the experimental method.

## 2. Resistance Sensitive Performance Test of Geogrid

### 2.1. Material and Fabrication of Specimen

The main material selected for this study was polylactic acid (PLA), whose physical properties are shown in Table 1. Based on previous research [34], the geogrid with distributed deformation monitoring function prepared by PLA as the main material can not only isolate the sensor from the contact with water in the external environment, but also the tensile strength of the geogrid sample prepared by this material is relatively higher than that of other materials, so this material is selected. The shells of geogrid were made of PLA. Conductive polymer was composed of 75% PLA powder, 5% carbon fiber power, and 20% PBS powder composited, in which PLA powder was used as the matrix, carbon fiber power as the conductive filler, and PBS powder composited as the toughening agent. The resistivity of conductive PLA material is 6.73 Ω.

**Figure 1:**
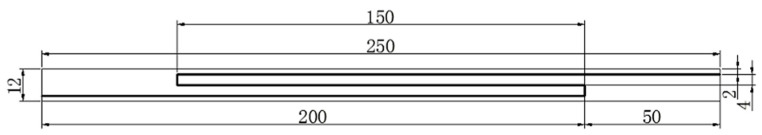
Layout of conductive wire inside the rib.

Geogrid is one of the main geosynthetics devices. A dual-nozzle 3D printer was used to produce geogrid specimens. One extruded nonconductive material is used as the main part of geogrid specimens, and the other extruded conductive material is used as the sensitive element embedded in shells. The printing accuracy was 0.4 mm, the printing speed was 45 mm/s, and the filling relative density was 100%. The print path of the conductive polymer with a diameter of 2 mm was folded three times; the conductive polymer print path was a longitudinal conductive polymer, folded three times in the longitudinal direction as shown in Figure 1.

**Figure 2:**
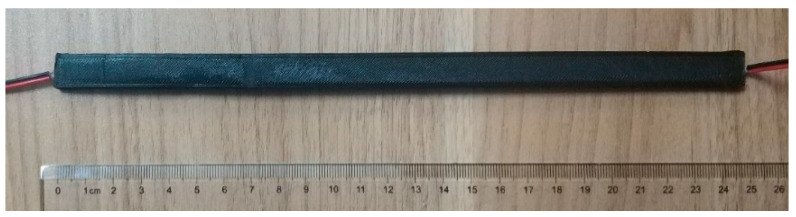
Single rib specimen.

**Figure 3:**
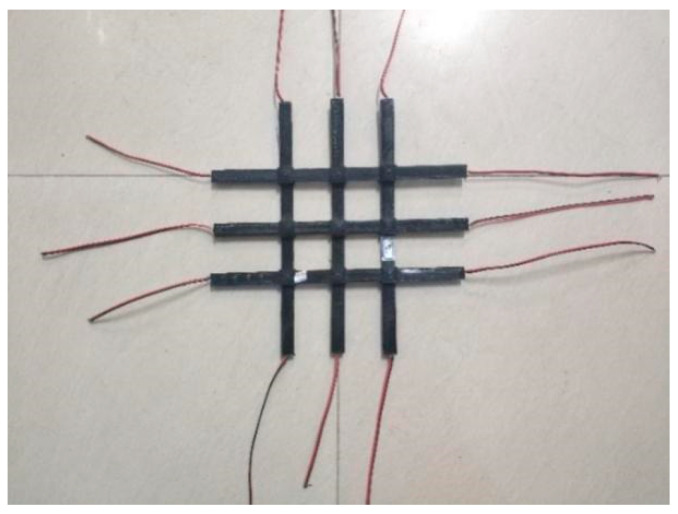
Schematic diagram of geogrid specimen.

The ribs of the geogrid were 250 mm long, 12 mm wide, and 3 mm thick, and wires were welded into both ends of the conductive polymer to record the resistance of both ends of the rib, as shown in Figure 2. Plastic connectors were also printed using PLA material. The ribs were crossed into them, the interval between each two ribs was 50 mm, and then connected to form a complete 3 × 3 geogrid test piece, as shown in Figure 3.

**Figure 4:**
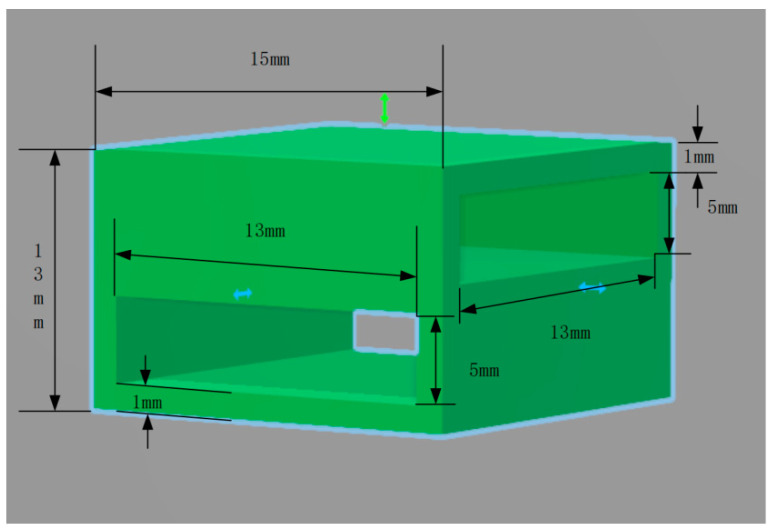
Nodal stereogram.

In addition, PLA material was used to print binding nodes (as shown in Figure 4), and ribs were inserted horizontally and longitudinally into the binding nodes for bonding, forming an integral geogrid specimen.

### 2.2. Experiment Procedure

A geo-bolt is a strip of geogrid. Tensile experiments have been used to test the sensitivity of geo-bolt to strain, and establish the relationship between their electrical resistance and strain or stress. The ribs used in this paper have also been tested for conductivity in the preliminary study and its sensing effect has been verified [34]. In this study, to verify the distributed deformation monitoring function of a geogrid in the two-dimensional direction, the loading experiments are carried out on the geogrid specimen.

**Figure 5:**
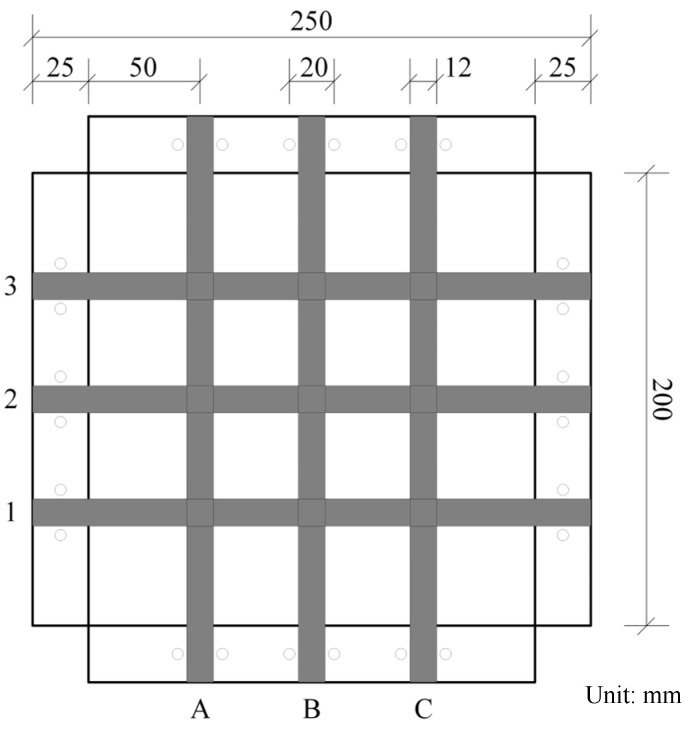
Size of the metal box.

**Figure 6:**
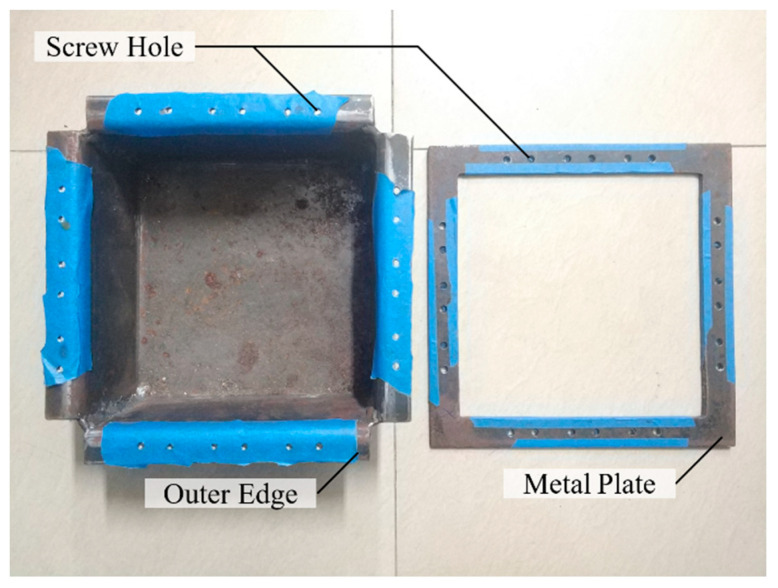
Metal box and metal plate.

**Figure 7:**
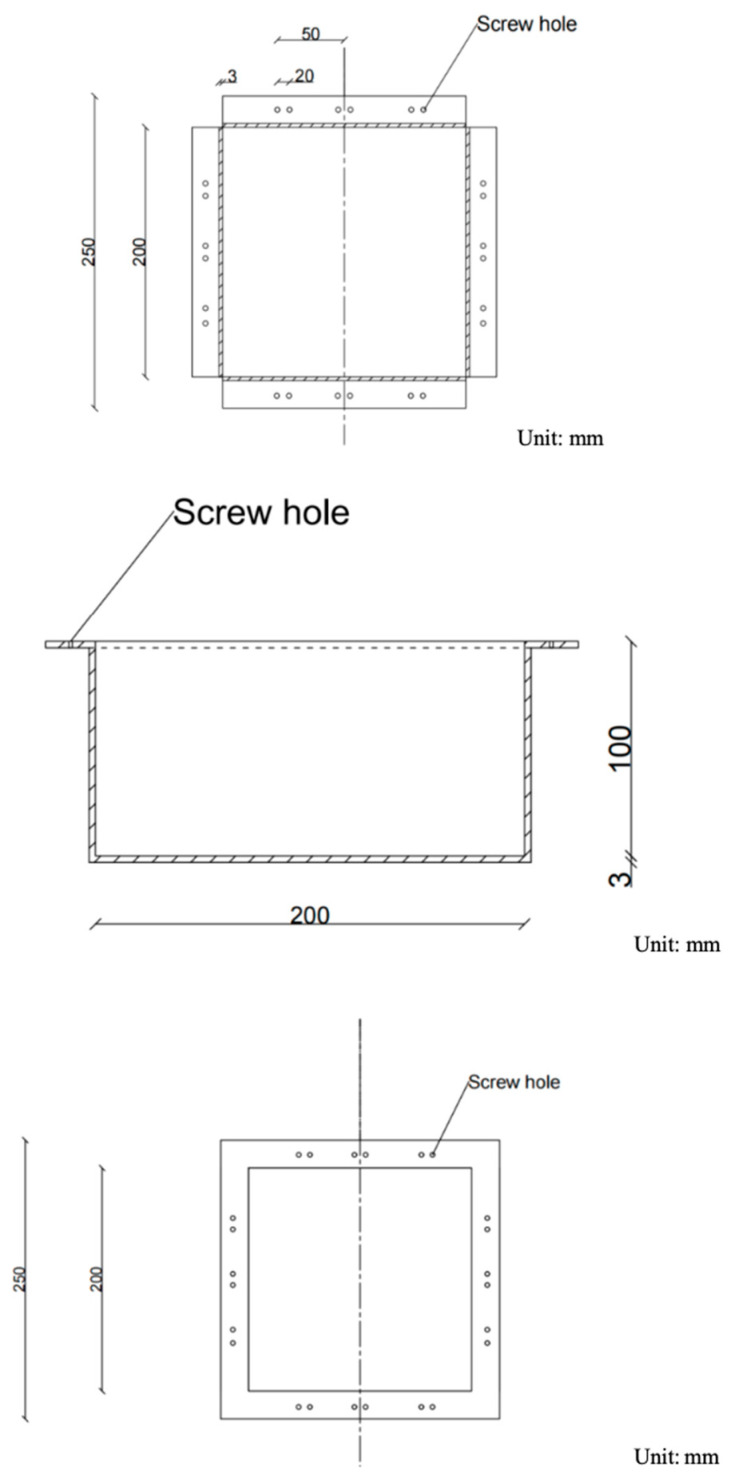
Three views of the metal box.

As shown in Figure 5, a special metal box is designed to fix the specimen, whose length, width, and height are 200 mm, 200 mm, and 100 mm, respectively. The top of the box extends outwards by 25 mm, forming four outer edges perpendicular to the sides. Screw holes with a diameter of 5 mm are set symmetrically on both sides of the trisection points of each outer edge, and the hole distance is 20 mm. In addition, a metal frame of 250 mm × 250 mm in size and 25 mm in width with screw holes corresponding to the outer edge of the box body are selected to cooperate with the metal box, as shown in Figure 6. The three views of the metal box are shown in Figure 7.

In this study, 9 grid nodes are selected as load bearing points, they are used as loading points. To identify them, the longitudinal ribs are numbered as A, B, and C, and the horizontal ribs are numbered as 1, 2, and 3, as shown in Figure 5. Therefore, loading points can be marked as 1A, 1B, 1C, and so on.

**Figure 8:**
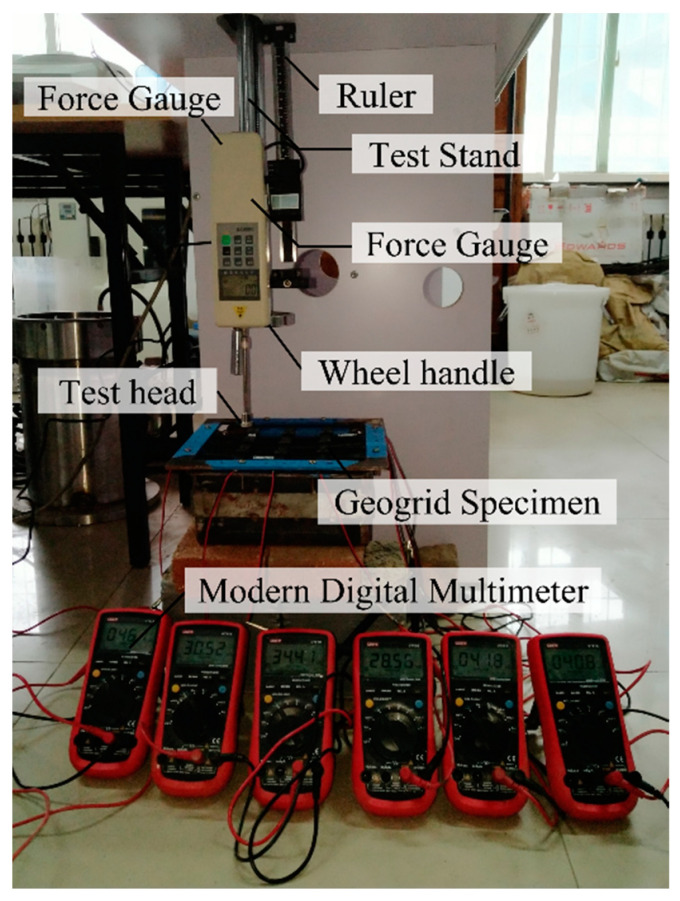
Experimental apparatus.

A digital force gauge is used for loading and the load value is manually adjusted with a wheel handle. Before the experiment, the digital force gauge is installed on the special test stand, which is fixed on the test bench in reverse. The geogrid specimen is placed on the metal box, so that all ribs are aligned with the three equal points of the metal outer edge, cover the metal frame, align with the upper and lower screw holes, tighten the screws, and fix the geogrid specimen in the metal box. Wires at each end of the geogrid rib are connected to multimeters to read the initial resistance. The length of rib is longer than the metal plate, as well as the wires are wrapped with insulating material. So, wires welded to both ends of the ribs will not contact the metal plate. With the wheel handle, load from 0 N to 100 N was applied to each loading point at an interval of 10 N and unloaded to 0 after reaching the peak load. Different loading points are tested in turn by adjusting the position of the metal box. The picture of the experimental device is shown in Figure 8.

## 3. Experimental Results Analysis

In the experiment, the resistance value of the whole rib is measured, which needs to be further processed to realize the function of distributed deformation monitoring. The analysis is carried out from three aspects: (1) studying the resistance variation of all ribs under local force; (2) further processing the data to obtain the resistance distribution in the plane; (3) studying the effect of fixed end on the resistance peak value.

### 3.1. Variation Rules of Resistance Values

**Figure 9:**
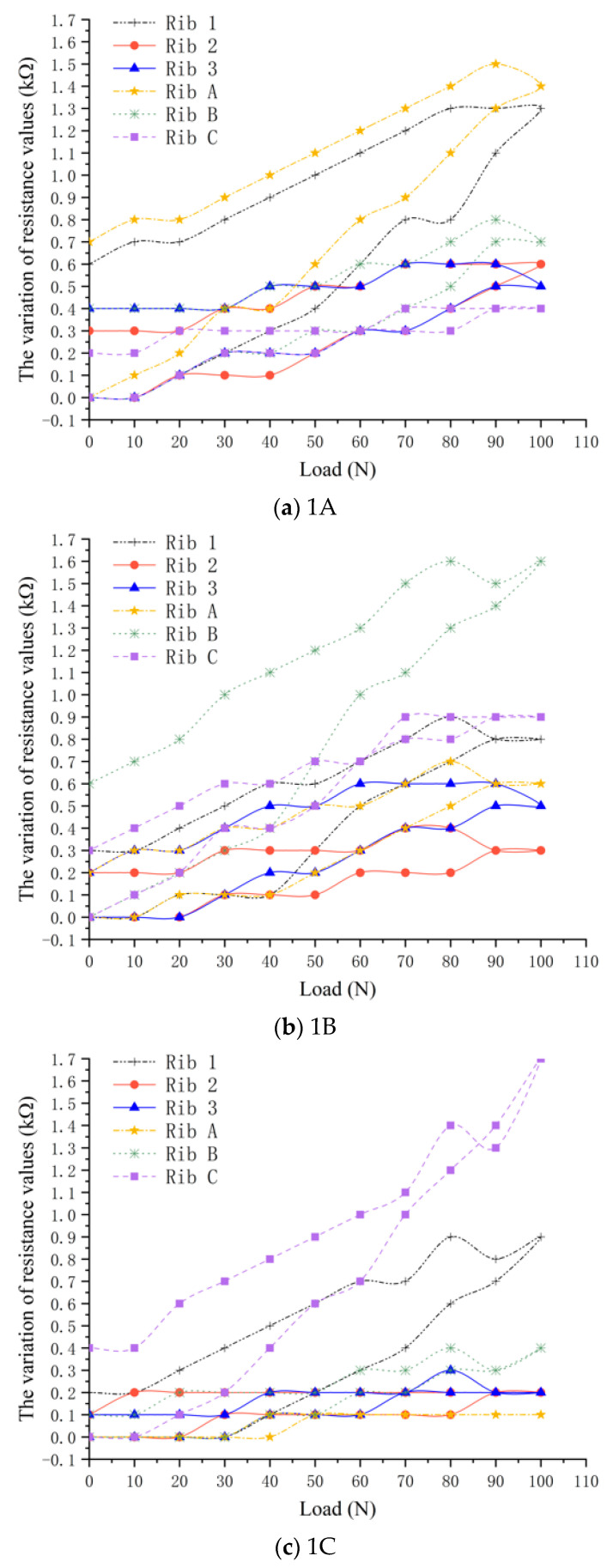

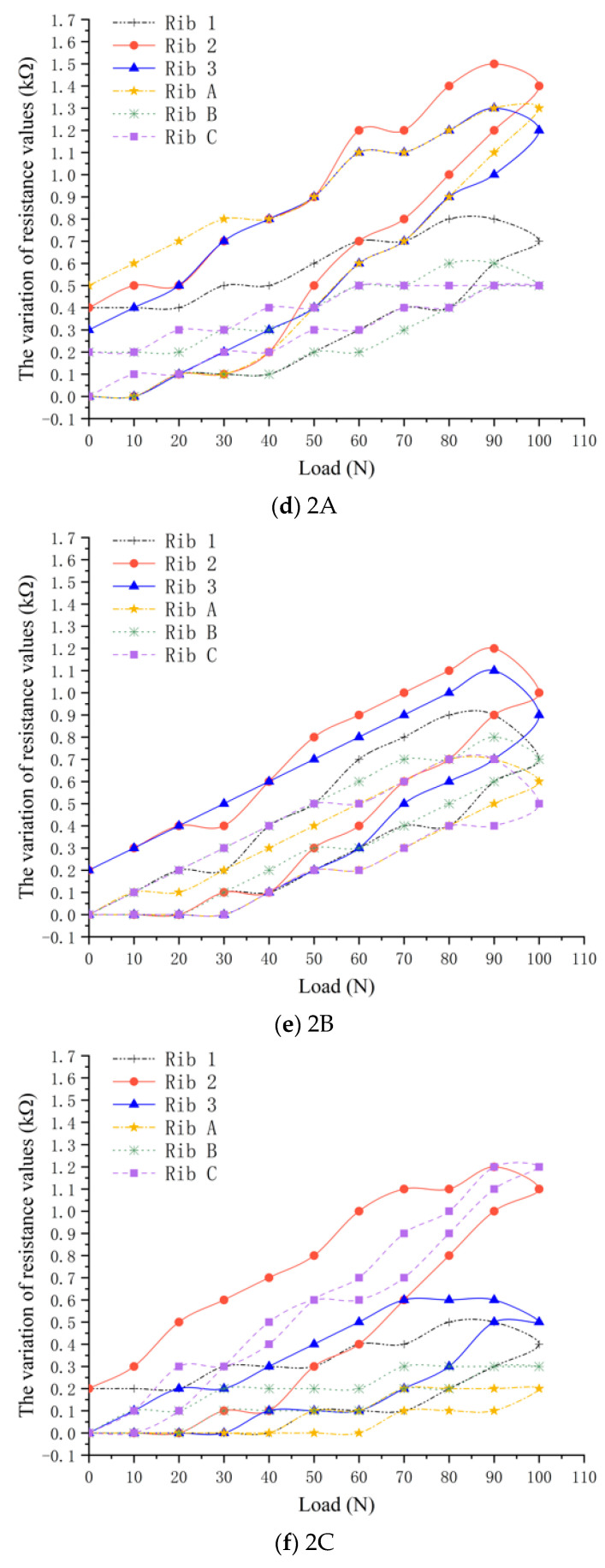

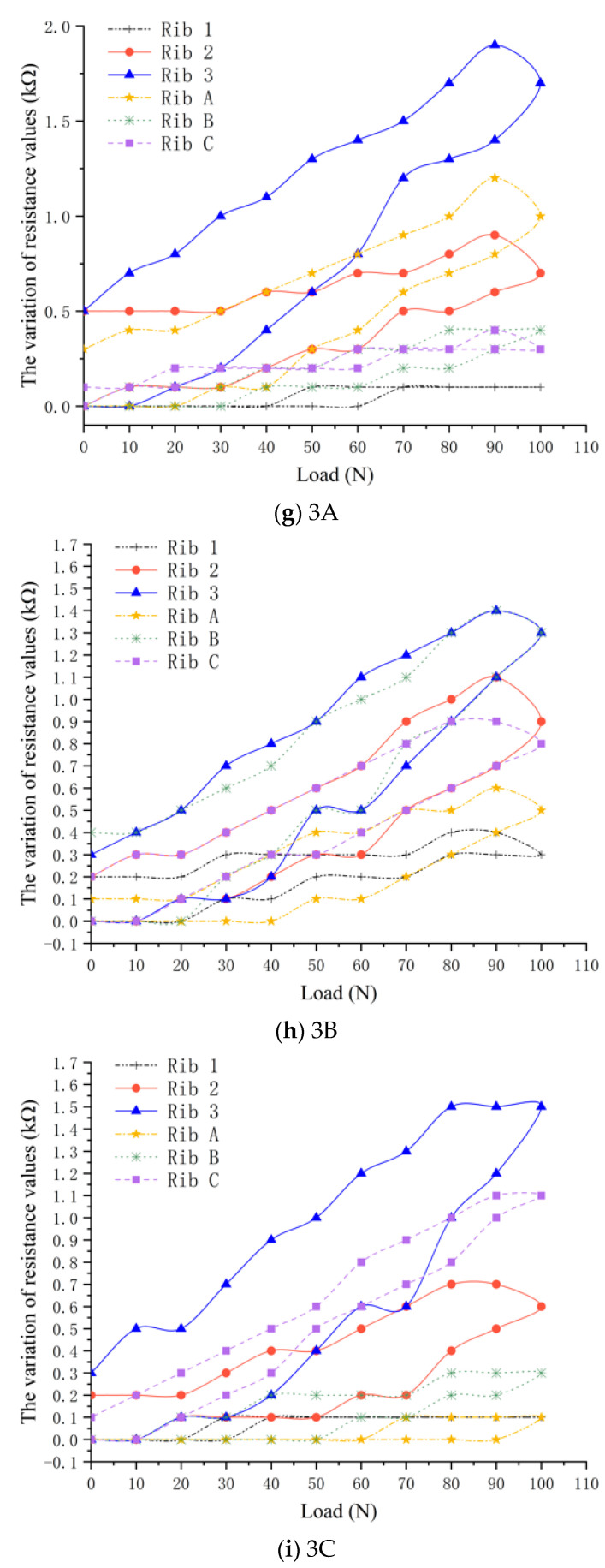
The variation of resistance values in each loading-unloading process.

In order to study the change rule of resistance of all position ribs under local stress, the loading–unloading test was carried out on nine measuring points, and the respective resistance values of six ribs in the loading–unloading process were recorded, and the data were analyzed and processed. The resistance changes of each rib during loading and unloading processes are shown in Figure 9.

Figure 9 indicates that when a load is applied to the loading point, the variation of resistance value of the two crossed ribs located at the loading point is significantly greater than that of other ribs. For the ribs arranged in the same direction, the closer the ribs to the force point, the greater the electrical resistance changes. For example, Figure 9c represents the status of each rib when applied to 1C loading point, the curves shows that the resistance values of ribs 1 and C changes most obviously, and their peak values of the resistance are significantly larger than other ribs. The resistance changes values of horizontal ribs decrease from rib 1 to rib 3, and meanwhile, the resistance changes values of longitudinal ribs decrease from rib C to rib A. The relationship between resistance value and stress state when other measuring points are stressed is shown in Figure 9. The results of the other eight groups of experiments also generally follow the same rule.

The curves of each single rib in Figure 9 show that during the loading–unloading process, the resistance value increases during the loading process, and values of resistance significantly decrease during the unloading process. However, when load was applied to 0 N, the resistance value does not go back to the initial value but is slightly larger than the initial value. As shown in Table 2, this is due to the plastic deformation of the ribs during loading, and the deformation cannot completely disappear after unloading.

### 3.2. Locating Deformation Position by the Variation of Resistance Values

It is difficult to visually detect internal deformation within the soil. Therefore, the primary objective of this study is how to use the resistance variation of the grating to monitor the internal surface area of the rock and soil mass, and to accurately locate the deformation position and deformation value. As an example, the resistance values measured in each rib when a 100 N load was applied to point 2C are presented in Table 3. In this study, the resistance value of each whole rib was measured when the wires are welded at both ends of the rib, and the deformation along the rib cannot be known. So, the resistance data of ribs in two directions should be integrated to obtain the resistance distribution on the geogrid plane, and the deformation monitoring of any local area can be realized.

At the position of the geogrid node, the vertical displacement of the node causes the deformation of two orthogonal ribs, resulting in resistance change. Therefore, the resistance change values of the two ribs are added together to represent the resistance change value at the node. The data at the edge of the geogrid in this experiment are treated based on the following assumptions:

(1) Points on the edge. Since each point on the edge of the metal frame involves the deformation of only one rib, the resistance change value on the corresponding rib is applied here.
(2) Corner points. At the four corner points, although no rib is installed here in this experiment, according to the analysis in the previous section, that is, the farther away the rib resistance is from the stress point, the smaller the change in the resistance will be. The distance between the four corner points and the stress point must be greater than the distance between the two adjacent edge points and the stress point, so the smaller change in the resistance of the two adjacent edge points can be used to simply replace the actual value at the corner point.

For the convenience of expression, the fixed position of both ends of horizontal ribs 1, 2, and 3 were marked as 0 and 4, respectively, and resistance data were marked every 50 mm. Similarly, the fixed positions of both ends of longitudinal ribs A, B, and C were marked as N and M, then upward every 50 mm there were resistance peak data, and these results were taken in turn. The variation values of resistance on the geogrid plane are shown in Table 3 (taking 2C loading point as an example). Table 2, below, takes 2B, 3A, and 3B as examples to show the results. It is worth mentioning that the displacement in Table 2 represents the longitudinal displacement of nodes.

**Figure 10:**
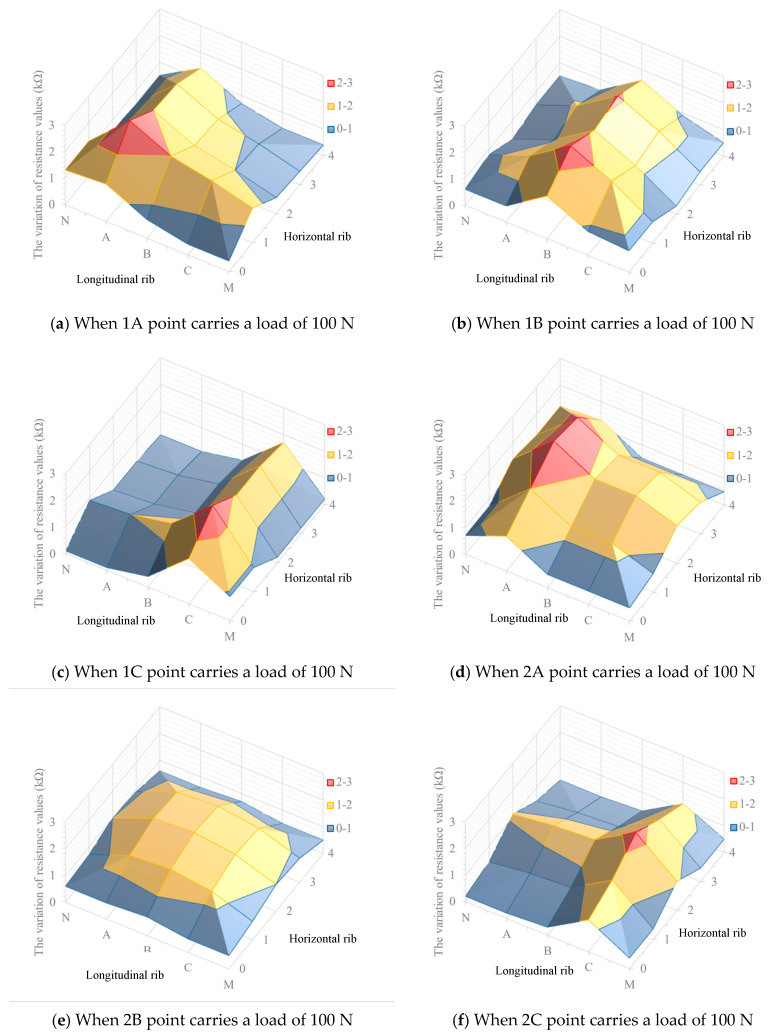

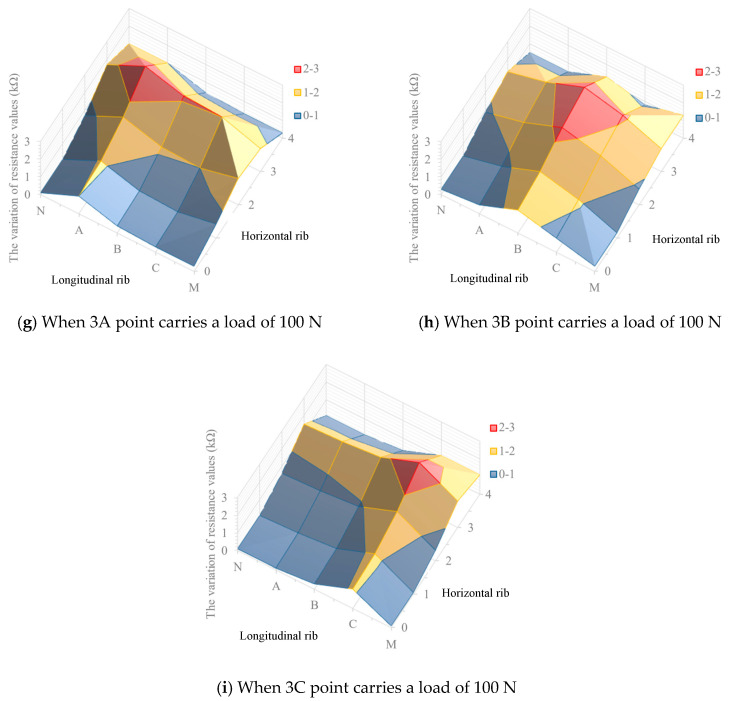
The distribution of resistance values of geogrid plane.

As shown in Figure 10f, when a load of 100 N was applied on 2C loading point, the geogrid formed three regions according to the variation of electrical resistance. The first is the red region, with the resistance changes between 2 and 3 kΩ, mainly near the intersection of rib 2 and rib C. The second is the yellow region, with the resistance changes between 1 and 2 kΩ, mainly distributing in the adjacent areas of rib 2 and rib C. The third is the blue region, with the resistance changes between 0 and 1 kΩ, appearing in the remaining region. The distributions of resistance values of geogrid plane when the load is applied on the other eight points also meet the same rule, as shown in Figure 10. In practical application, when the internal deformation of soil structure changes the stress situation of the geogrid, the deformation position can be locked by the variation of electrical resistance.

As shown in Table 4, with the increase of load, settlement displacement of the force point increases gradually, and electrical resistance of geogrid increases. So, the relationship between the variation of electrical resistance and settlement displacement could be established in the practical application. The magnitude of settlement displacement can be inversely deduced by the variation of electrical resistance, and whether the internal deformation of soil is close to the critical value can also be estimated.

### 3.3. Effect of Loading Position on Variation of Electrical Resistance

Figure 10 indicates that the characteristics of geogrid resistance changing with load are different due to different loading positions. When 1C, 1B, and 2B are applied loads of 100 N, the variation of electrical resistance of two corresponding ribs in the horizontal and longitudinal directions are analyzed. The points 1B, 1C, and 2B represent the loading points near two fixed ends, one fixed end, and the central position, respectively.

**Figure 11:**
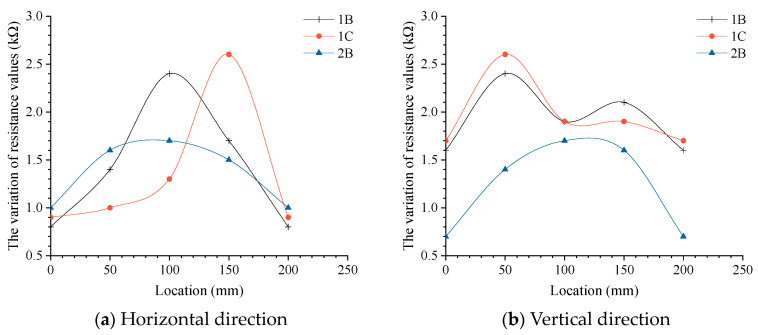
The variation of resistance values of profiles.

The three curves shown in Figure 11a represent the variation of electrical resistance at the longitudinal section of the corresponding horizontal rib when a load of 100 N is applied to 1B, 1C and 2B loading points. When the load level of the three points is the same, the variation of electrical resistance caused by the load acting on the loading point (1C) near the two fixed ends is the largest, the variation of electrical resistance caused by the load acting on the loading point (1B) near one fixed end is the second, and the variation of electrical resistance caused by the loading point (2B) acting on the central position is the smallest.

The three curves shown in Figure 11b represent the variation of electrical resistance at the longitudinal section of the corresponding vertical rib when a load of 100 N is applied to 1B, 1C, and 2B loading points. When the load level of the three points is the same, in descending order of the resistance change caused by load are also 1C, 1B, and 2B. It would be expected that changes in the electrical resistance would be greater at a central point that typically undergoes greater deformation under a given load as compared to a point near a boundary. However, the opposite trend has been observed in this research, which may be due to the undesirable boundary effects (the presence of a rigid frame) that have influenced the experiments.

## 4. Simulation and Analysis

In addition, the geogrid model is numerically simulated, and the experimental results are compared with the numerical results to verify the experimental findings.

### 4.1. Setup of Numerical Simulation

In order to simulate the force of geogrid in soil closer to the actual scale, the 3 × 3 model in the above experiment is extended based on the finite element analysis software ANSYS. Under normal conditions, the stress on the geogrid is much lower than its tensile strength, so the Shell63 unit and the line elasticity simulation is selected. The physical property parameters of PLA material are selected: the density, elastic modulus, and Poisson’s ratio are 1240 kg/m^3^, 3000 Mpa, and 0.36, respectively. The geogrid is modeled in with mesh size of 2, and a total of 3276 meshes are used. The fixed support boundary condition is adopted at edges of ribs. The loading area is 12 × 12 = 144 mm^2^ (the area of grid node).

### 4.2. Stress Variation of Geogrid Model

The nine loading points are divided into three categories: the center point, point near one fixed end, and point near two fixed ends. Three measuring points, 2B, 3B, and 3C, were taken as representative values of the numerical simulation, and 0–100 N force was applied to one measuring point and recorded every 10 N, and the internal stress value was recorded every 10 mm on the length of a rib of 200 mm. A total of 21 locations were recorded. Due to stress concentration, the value of the side near the stress measurement point is discarded, and the stress value of the root rib under a certain force is averaged, and the corresponding broken line diagram is drawn to compare with the experimental results.

**Figure 12:**
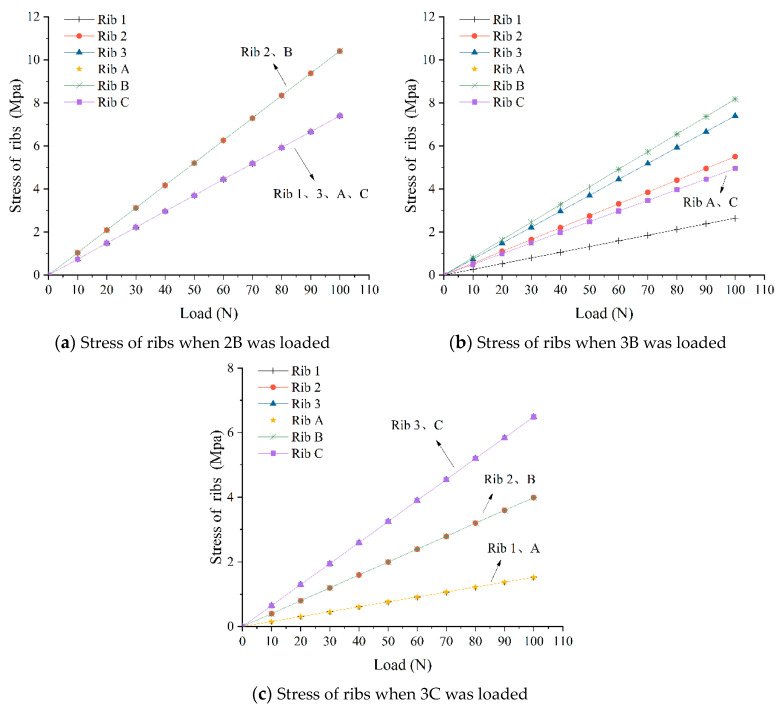
The variation of stress of the ribs in numerical simulation.

The trend of load–stress changes of three loading points are shown in Figure 12. In the numerical simulation, when the load on a loading point increases gradually, the internal average stress of each rib increases linearly. As shown in Figure 12, the change of the internal average stress of the two ribs symmetrical to the loading point is almost the same. Under the same external force, the closer to the loading point, the greater the internal average stress value of the rib, and the farther away from the loading point, the smaller the internal average stress value of the rib.

In the laboratory loading experiments, the variation of electrical resistance on the geogrid plane has similar rules, but the resistance changes of ribs in symmetrical position are not identical. The reason is that the distribution of conductive fillers in conductive polymer is not uniform due to the immaturity of the specimen manufacturing process.

### 4.3. Stress Distribution on Geogrid

The stress distribution of the geogrid plane when a load is applied on a loading point can be obtained through numerical simulation and compared with the electrical resistance distribution measured by experiment. The loading points 2B, 3B, and 3C were selected as representative points, which is due to the symmetrical distribution of nine loading points in the resistance sensitivity performance test of geogrid.

**Figure 13:**
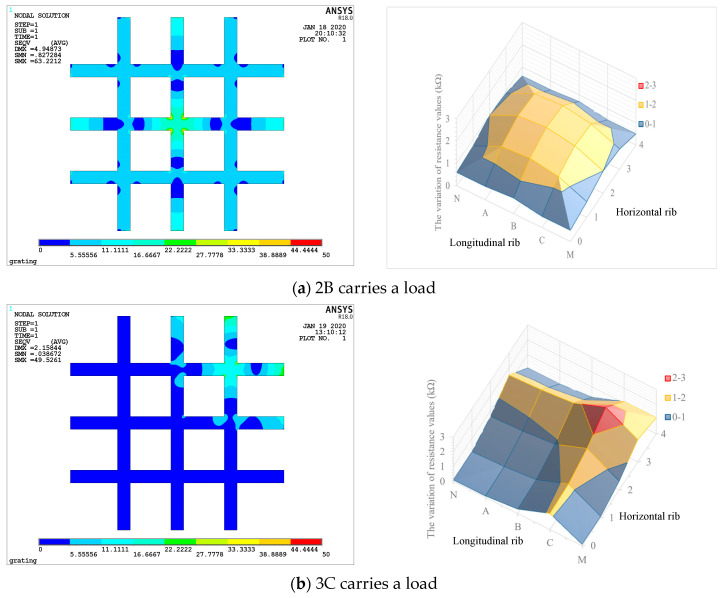

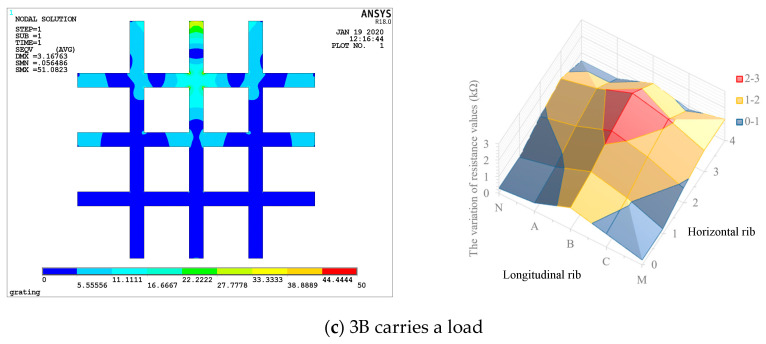
Comparison of resistance and stress values on geogrid plane.

Figure 13 shows the distribution of stress on geogrid plane when 2B, 3B, and 3C loading points are applied a load of 100 N. It can be compared with the electrical resistance distribution measured by experiment shown in Figure 10. The distribution of resistance in Figure 10 is relatively consistent with the distribution of stress in Figure 13, which can roughly reflect the internal stress situation of geogrid rib. For 2B, the peak value of resistance change is relatively consistent with the variation range of stress in numerical simulation, which basically covers all measuring points of the whole geogrid. For 3C, the peak range of resistance is basically concentrated around the loading point. In the numerical simulation, part of the peak value of stress extends to the adjacent points. It may be due to the small change of displacement near the boundary on both sides, which is not enough to cause the change of resistance. For 3B, four measuring points are covered by the peak range of resistance change, which is basically consistent with the results of numerical simulation stress change.

According to the data in Table 2, the settlement at point 2B is greater than that at points 3C and 3B. Theoretically, the greater the elongation, the greater the change of electrical resistance. Therefore, when the same load is applied at three loading points, 3C, 3B, and 2B, the change of resistance of rib caused by 2B should be greater than that of 3C and 3B. However, according to Figure 10, when the same load is applied at 3C, 3B, and 2B, the electrical resistance change of the ribs decreases in turn. A reasonable explanation for this contradictory result can be found in Figure 13. From the numerical simulation results, when the loading point is close to the edge or corner (such as Figure 13b,c), the stress concentration will occur at the fixed end of the rib, and the increase of local stress will cause a sharp decrease in the local electrical conductivity, which is reflected in the increase of the resistance value of the whole rib. Therefore, in the actual engineering application, the critical monitoring area should be placed in the center of the geogrid to increase the monitoring accuracy.

## 5. Conclusions

To study the distributed deformation monitoring function of a self-monitoring geogrid, loading–unloading experiments and numerical simulation were performed. Conclusions were obtained as follows:

1. Through the analysis and comparison of the results of the points at the crossing of horizontal and longitudinal ribs, it can be found that when the point is loaded, the resistance value change of the two ribs passing through the loading point is significantly higher than that of other ribs. Moreover, in the same direction (horizontal or longitudinal) of the ribs, the closer the rib is to the loading point, the greater the resistance change is, and the farther the rib is from the loading point, the smaller the resistance change is.
2. The resistance distribution of geogrid plane can be obtained by superimposing the resistance changes of the ribs in the horizontal and longitudinal directions of the loading points. According to the size of the resistance change, the position of the deformation in the rock mass and the size of the settlement displacement can be preliminarily determined.
3. By analyzing the resistance variation of each rib separately, during the loading–unloading process, it can be seen that resistance value increases during loading and decreases during unloading.
4. Through the analysis of the test results of the same load on the loading points in different positions, the peak resistance changes caused by the load on the loading points in different positions are different. The peak resistance changes near the fixed end are larger, while the peak resistance changes near the center are smaller.
5. The closer the loading point to the center position is to the external force, the greater the range of resistance changes, and the greater the possibility of being monitored. On the contrary, the closer the loading point to the boundary of the geogrid is to the external force, the smaller the range of resistance changes. Therefore, in practical engineering applications, the center position of the geogrid is suggested to be laid in the key monitoring area.
6. By comparing the plane stress distribution of geogrid after being stressed by numerical simulation with the plane resistance distribution measured by experiment, it can be concluded that reversing the deformation of the geogrid by using the variation of the resistance value of each rib is feasible.

## Figures and Tables

**Table 1:** Physical properties of PLA.

Material	Density (kg/m^3^)	Tensile strength (MPa)	Elongation at Break (%)
PLA	1.24	50 ± 1.3	2.0 ± 0.4

**Table 2:** Displacement and resistance values of representative points during loading–unloading process.

Load (N)	2B		3A		3B	
	Displacement (mm)	The Variation of Resistance Values (kΩ)	Displacement (mm)	The Variation of Resistance Values (kΩ)	Displacement (mm)	The Variation of Resistance Values (kΩ)
0	0	0	0	0	0	0
10	1	0	2	0	0.5	0
20	2	0	3	0.05	1	0.05
30	3.5	0.1	3.5	0.15	2	0.15
40	4.5	0.15	4	0.25	3	0.25
50	6	0.3	5	0.45	4	0.5
60	7	0.35	6	0.6	5	0.5
70	8	0.5	6.5	0.9	6	0.75
80	9	0.6	7	1	7	0.9
90	10	0.75	8	1.1	7.5	1.1
100	11	0.85	8.5	1.35	8	1.3
90	11	1	8	1.55	8	1.4
80	10	0.9	8	1.35	7.5	1.3
70	9.5	0.85	7	1.2	7	1.15
60	9	0.75	7	1.1	6	1.05
50	7.5	0.65	6	1	5	0.9
40	6	0.5	5	0.85	4	0.75
30	4.5	0.35	4	0.75	3	0.65
20	3	0.3	3	0.6	2	0.5
10	2	0.2	2	0.55	1	0.4
0	1	0.1	1.5	0.4	0	0.35

**Table 3:** The variation of resistance values of each rib when 2C point received a load of 100 N.

The number of the ribs	1	2	3	A	B	C
The variation of resistance values (kΩ)	0.4	1.1	0.5	0.2	0.3	1.2

**Table 4:** The variation of resistance (kΩ) values of each point when 2C point received a load of 100 N.

Point	N	A	B	C	M
0	0.2	0.2	0.3	1.2	0.4
1	0.4	0.6	0.7	1.6	0.4
2	1.1	1.3	1.4	2.3	1.1
3	0.5	0.7	0.8	1.7	0.5
4	0.2	0.2	0.3	1.2	0.5

## Data Availability

Data are contained within the article.
